# Quantitative Assessment of Cytosolic *Salmonella* in Epithelial Cells

**DOI:** 10.1371/journal.pone.0084681

**Published:** 2014-01-06

**Authors:** Leigh A. Knodler, Vinod Nair, Olivia Steele-Mortimer

**Affiliations:** 1 Paul G. Allen School for Global Animal Health, College of Veterinary Medicine, Washington State University, Pullman, Washington, United States of America; 2 Laboratory of Intracellular Parasites, Rocky Mountain Laboratories, National Institute of Allergy and Infectious Diseases, National Institutes of Health, Hamilton, Montana, United States of America; 3 Research Technologies Branch, Rocky Mountain Laboratories, National Institute of Allergy and Infectious Diseases, National Institutes of Health, Hamilton, Montana, United States of America; The Ohio State University, United States of America

## Abstract

Within mammalian cells, *Salmonella enterica* serovar Typhimurium (*S*. Typhimurium) inhabits a membrane-bound vacuole known as the *Salmonella*-containing vacuole (SCV). We have recently shown that wild type *S*. Typhimurium also colonizes the cytosol of epithelial cells. Here we sought to quantify the contribution of cytosolic *Salmonella* to the total population over a time course of infection in different epithelial cell lines and under conditions of altered vacuolar escape. We found that the lysosomotropic agent, chloroquine, acts on vacuolar, but not cytosolic, *Salmonella*. After chloroquine treatment, vacuolar bacteria are not transcriptionally active or replicative and appear degraded. Using a chloroquine resistance assay, in addition to digitonin permeabilization, we found that *S*. Typhimurium lyses its nascent vacuole in numerous epithelial cell lines, albeit with different frequencies, and hyper-replication in the cytosol is also widespread. At later times post-infection, cytosolic bacteria account for half of the total population in some epithelial cell lines, namely HeLa and Caco-2 C2Bbe1. Both techniques accurately measured increased vacuole lysis in epithelial cells upon treatment with wortmannin. By chloroquine resistance assay, we also determined that *Salmonella* pathogenicity island-1 (SPI-1), but not SPI-2, the virulence plasmid nor the flagellar apparatus, was required for vacuolar escape and cytosolic replication in epithelial cells. Together, digitonin permeabilization and the chloroquine resistance assay will be useful, complementary tools for deciphering the mechanisms of SCV lysis and *Salmonella* replication in the epithelial cell cytosol.

## Introduction

Intracellular pathogens have adopted diverse strategies in order to survive and proliferate within eukaryotic cells. Simplistically, these can be divided into two distinct categories (i) those that allow for survival within a membrane-bound vacuole or (ii) those that allow for access to the cytosol. The default pathway for microbes that are internalized by host cells is to be delivered to the lysosome for degradations. While certain bacteria have adapted to live within the degradative lysosomal environment, such as *Coxiella burnetii* [1], others either modify their vacuole or escape into the cytosol. Modification of the vacuole can involve arresting, or slowing, its maturation or its diversion from the endocytic pathway to another intracellular compartment. Examples are *Mycobacterium tuberculosis* [2] and *Brucella abortus* [3], respectively. Alternatively some microbes, such as *Legionella pneumophila* [4] and *Chlamydia trachomatis* [5], are sequestered into a compartment that is separated from the endocytic pathway but interacts with other organelles. Cytosolic pathogens, exemplified by *Shigella flexneri* and *Listeria monocytogenes*, escape early from the nascent vacuole and then replicate extensively within the cytosol [6].

Ingestion of the Gram-negative pathogen, *Salmonella enterica* serovar Typhimurium (*S*. Typhimurium), leads to self-limiting gastroenteritis in humans and a systemic typhoid-like disease in mice. In infected hosts, *Salmonella* colonizes several different cell types, including epithelial cells, dendritic cells and macrophages [7]–[9]. After their uptake into host cells, bacteria are contained within a modified phagosome called the *Salmonella*-containing vacuole (SCV) [10], [11]. SCV biogenesis is characterized by extensive interactions with the endocytic pathway and the mature SCV is rich in lysosomal membrane-proteins such as LAMP1 and LAMP2 [12]. Trafficking of the SCV and maintenance of vacuolar integrity is dependent upon the actions of numerous type III effectors translocated by either type III secretion system 1 (T3SS1) or T3SS2 [11], encoded on *Salmonella*-pathogenicity island-1 (SPI-1) and SPI-2, respectively.

Despite being historically classified as a vacuolar pathogen, it is now well-documented that a proportion of intracellular *S*. Typhimurium lyse the nascent SCV and escape into the cytosol of epithelial cells [13]–[15]. Autophagy reportedly limits the cytosolic proliferation of *S*. Typhimurium after this lytic event [13], [14], [16]. Contrary to this, *Salmonella* deficient for the type III effector, *sifA*, cannot maintain their vacuolar integrity and subsequently proliferate in the cytosol of epithelial cells ≥6 h post-infection (p.i.) [17]. Cytosolic Δ*sifA* bacteria are not detected by autophagy [13]. Furthermore, we have recently shown that wild type *Salmonella* can replicate to vast numbers in epithelial cytosol at late times p.i., which we have termed hyper-replication (defined as ≥100 bacteria/cell) [18], suggesting that autophagic control of cytosolic *Salmonella* may only be an early, transient event.

Studies in cultured epithelial cells have shown that approximately 10% of infected cells contain hyper-replicating *Salmonella* at 8 h p.i. [18], [19]. But what proportion of the total bacterial population is vacuolar versus cytosolic? To answer this question, here we have applied two independent techniques, digitonin permeabilization and a chloroquine (CHQ) resistance assay, to quantify the bacteria occupying these different subcellular localizations under various infection conditions. Our data establish that cytosolic *Salmonella* constitute a significant proportion of the total bacterial population in epithelial cells.

## Materials and Methods

### Bacterial Strains and Plasmids

*Salmonella enterica* serovar Typhimurium SL1344 was the wild-type strain used in this study [20]. The Δ*ssaR*, χ3340, Δ*sifA*, *flgB*::Tn10, ΔSPI1::kan and *invA*::kan strains have been described previously [21]–[26]. The “effectorless” mutant, SB1011, is deleted or carries loss-of-function mutations for seven type III effectors, *sptP*, *sopE*, *sopE2*, *sopB*, *avrA*, *sopA* and *sipA* and was provided by Dr J. Galán (Yale University). *prgI*::kan was constructed in *S*. Typhimurium SL1344 using λ Red recombinase technology [27] with the oligonucleotides prgI-KO-F (5′ ACT TTA ATT TAA CGT AAA TAA GGA AGT CAT TAT GGC AAC ACC TGT AGG CTG GAG CTG CTT CG 3′) and prgI-KO-R (5′ CTG CCC TAT AAC GGC ATT CTC AGG GAC AAT AGT TGC AAT CGA CAT ATG AAT ATC CTC CTT AG 3′). The following plasmids have been described: pJC45, a plasmid encoding anhydrotetracycline (ATc)-inducible green fluorescent protein (GFPmut3) [28], pFPV-mCherry encodes mCherry under the control of the *rpsM* promoter [29], pMPMA3ΔPlac-P*prgH*-GFP[LVA] encodes destabilized GFP under the control of the *prgH* promoter [24].

### Chemicals and Reagents

Rat tail collagen I was from BD Biosciences (San Jose, CA). CHQ, transferrin, digitonin, saponin and sodium deoxycholate (DOC) were from Sigma-Aldrich (St Louis, MO). ATc was from Acros Organics (Thermo Fisher Scientific, Pittsburgh, PA). Wortmannin (WTM) was from Calbiochem (EMD Millipore Chemicals, Billerica, MA). Antibodies for immunofluorescence were: rabbit anti-*Salmonella* lipopolysaccharide (LPS) (O-antigen Group B Factors 1, 4, 5, 12; BD Difco) and mouse anti-human LAMP1 (clone H4A3, developed by J.T. August and obtained from the Developmental Studies Hybridoma Bank (DSHB) developed under the auspices of the National Institute of Child Health and Human Development and maintained by the University of Iowa, Department of Biological Sciences, Iowa City, IA). Alexa Fluor 488, 568 or 647 goat anti-rabbit or goat anti-mouse IgG secondary antibodies, normal goat serum (NGS) and Hoechst 33342 were from Life Technologies (Grand Island, NY).

### Mammalian Cell Lines

All epithelial cell lines were purchased from American Type Culture Collection (ATCC) and used within 15 passages of receipt. HeLa cervical adenocarcinoma cells (ATCC CCL-2) and HuTu 80 duodenal adenocarcinoma cells (ATCC HTB-40) were grown in Eagle’s modified medium (EMEM, Corning cellgro®, Manassas, VA) containing 10% (v/v) heat-inactivated fetal calf serum (HI-FCS, Invitrogen, Carlsbad, CA). Caco-2 C2Bbe1 colorectal adenocarcinoma cells (ATCC CRL-2102) were grown in Dulbecco’s modified Eagle’s medium (DMEM, Corning cellgro®) containing 0.01 mg/ml transferrin and 10% (v/v) HI-FCS. HCT 116 colorectal carcinoma cells (ATCC CCL-247) were grown in McCoy’s 5a modified medium (Corning cellgro®) containing 10% (v/v) HI-FCS. Cells were seeded in 24-well tissue-culture treated plates (Corning Costar®) 18–24 h prior to infection. Seeding densities were 5×10^4^ cells/well (HeLa), 6×10^4^ cells/well (C2Bbe1), 1.2×10^6^ cells/well (HCT 116) and 8×10^4^ cells/well (HuTu 80). For immunofluorescence, cells were seeded on acid-washed glass coverslips (Fisherbrand) in 24-well plates 18–24 h prior to infection. Seeding densities were 6×10^4^ cells/well (HeLa), 4–5×10^4^ cells/well (C2Bbe1), 1.2×10^5^ cells/well (HCT 116) and 9×10^4^ cells/well (HuTu 80). C2Bbe1 and HCT 116 cells were seeded on collagen-coated wells or coverslips to promote adherence.

### Bacterial Infections

Bacteria were grown in LB-Miller broth (BD Difco) to late log-phase as described [24], then centrifuged at 8,000×*g* for 2 min and resuspended in Hank’s buffered saline solution (HBSS, Corning cellgro®). Bacteria were added to epithelial cells at a multiplicity of infection (MOI) of 50–100 for 10 min at 37°C. For the chloroquine resistance assay in C2Bbe1 cells, the MOI was increased to ∼1,000 for *prgI*::kan, *invA*::kan, ΔSPI1::kan and “effectorless” mutant bacteria to facilitate bacterial entry. Non-internalized bacteria were removed by three washes in HBSS, and cells incubated in growth media until 30 min p.i. Then growth media containing 100 µg/ml gentamicin was added for 1 h, followed by growth media containing 10 µg/ml gentamicin for the remaining incubation time. For enumeration of intracellular bacteria, epithelial monolayers were washed once in phosphate-buffered saline (PBS), then solubilized in 1 ml 0.2% (w/v) DOC in PBS and serial dilutions plated on LB agar. WTM (100 nM) was added to epithelial cells from 45 min prior to infection to 90 min p.i., whereupon it was washed out and infection continued as described above.

### Chloroquine (CHQ) Resistance Assay

To quantify the proportion of cytosolic bacteria in the total population, we used a CHQ resistance assay [30]–[32]. Epithelial cells were infected in 24-well plates as described above. For each timepoint, two wells were incubated in the presence of CHQ and gentamicin for 1 h (CHQ-resistant bacteria) and another two wells were incubated with gentamicin only (total bacteria). Infected cells were solubilized in DOC as described above and the numbers of viable bacteria were determined by plating serial dilutions on LB agar. CHQ concentrations were titrated for each cell line to obtain maximal vacuolar killing of bacteria without compromising cell viability and loss. CHQ concentrations used were: HeLa, 400 µM; HCT 116, 400 µM; HuTu 80, 200 µM; and Caco-2 C2Bbe1, 800 µM.

### Immunofluorescence

Immunofluorescence staining was as described previously [24]. To identify cytosolic bacteria by fluorescence microscopy, we used a digitonin permeabilization assay to deliver anti-LPS antibodies directly to the cytosol of epithelial cells [18], [19], [33]. Optimal digitonin concentrations were determined for each epithelial cell line according to two parameters, maximal plasma membrane permeabilization with minimal cell detachment from the glass coverslips. Digitonin concentrations were: (i) HeLa, 45 µg/ml for 1 min; (ii) HCT116, 50 µg/ml for 1 min; (iii) HuTu 80, 25 µg/ml for 1.5 min. This assay could not be applied to Caco-2 C2Bbe1 cells because of their detachment from coverslips at digitonin concentrations that were insufficient for permeabilization.

### Transmission Electron Microscopy

*Salmonella*-infected HeLa cells grown on Thermanox® coverslips (Ted Pella, Inc., Redding, CA) were fixed with 2.5% glutaraldehyde in 0.1 M sodium cacodylate buffer (Electron Microscopy Sciences, Hatfield, PA). All subsequent processing steps were carried out in a Pelco Biowave laboratory microwave system (Ted Pella, Inc.) at 250 W. Following fixation, the monolayer was rinsed with buffer and post fixed with 1% osmium tetroxide reduced with 0.8% potassium ferrocyanide in 0.1 M sodium cacodylate buffer under 20 in. Hg vacuum. After rinsing in 0.1 M sodium cacodylate buffer, the monolayer was treated with 1% aqueous tannic acid and *en bloc* stained using 1% aqueous uranyl acetate under vacuum. The cells were then rinsed with distilled water and dehydrated in a gradient ethanol series. The monolayer was infiltrated under vacuum with 1∶1 (ethanol: Spurr’s resin) and 100% resin. The cells were later embedded in resin and sectioned on a UC6 ultramicrotome (Leica Microsystems, Vienna, Austria). Sections were collected on a 200 mesh copper grid, stained for contrast using 4% uranyl acetate and lead citrate prior to imaging on a Tecnai BioTwin Spirit TEM (FEI, Hillsboro, OR). Digital images were acquired with a Hamamatsu Orca digital camera system (AMT, Danvers, MA.).

## Results

### Cytosolic, Invasion-primed Salmonella Occur in Multiple Epithelial Cell Lines

**Figure 1:**
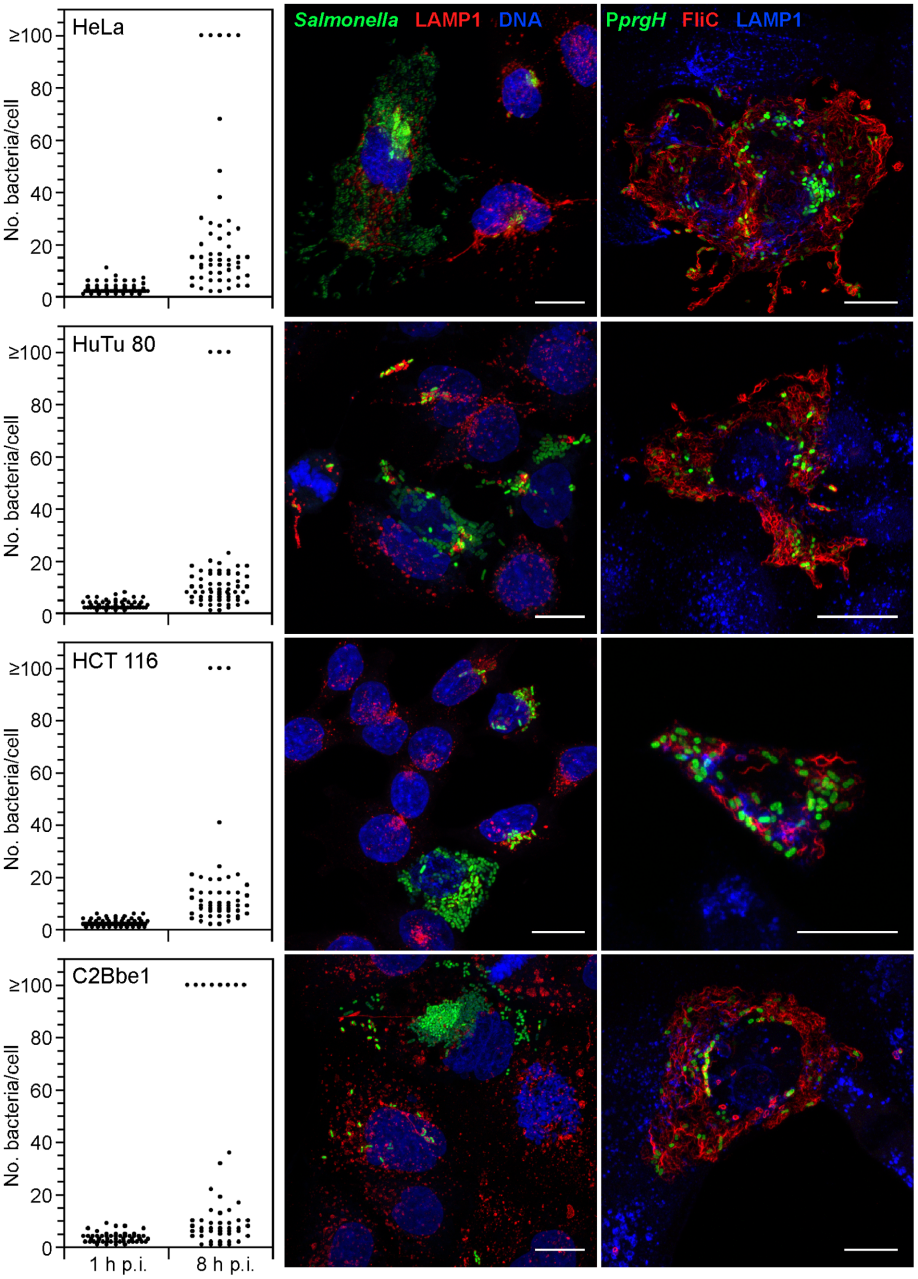
Hyper-replicating invasion-primed *Salmonella* occur in numerous epithelial cell lines. Epithelial cells were infected with mCherry *S.* Typhimurium (left and middle panels) or *S.* Typhimurium harboring a reporter plasmid, P*prgH*-GFP[LVA] (right panels). Left panel; cells were fixed at 1 h and 8 h p.i. and the number of internalized bacteria per cell was scored by fluorescence microscopy. Each dot represents one infected cell (≥50 infected cells were scored for each timepoint). Data are from one experiment representative of at least three independent experiments. Middle and right panels; representative confocal images of hyper-replicating, cytosolic *Salmonella*. Cells were fixed at 8 h p.i., permeabilized and immunostained for the vacuolar membrane marker, LAMP1 (middle panels), and flagellin, FliC (right panels). DNA was stained with Hoechst 33342. Scale bars are 20 µm.

We have previously reported the presence of hyper-replicating *Salmonella* in the cytosol of the polarized intestinal epithelial cell line, C2Bbe1 [18]. To investigate whether hyper-replication is a widespread phenomenon in tissue culture epithelial cells, we compared the progression of bacterial infections in non-polarized C2Bbe1 cells with HeLa cells, which have been used extensively to decipher the intracellular trafficking of *Salmonella*, and two intestinal epithelial cells lines, HuTu 80 and HCT 116. Cells were infected with *Salmonella* constitutively expressing mCherry (mCherry *Salmonella*), fixed at 1 h and 8 h p.i. and the number of intracellular bacteria per cell scored by fluorescence microscopy. For all cell lines, there was variation in the number of internalized bacteria, ranging between 1 and 10 bacteria per cell at 1 h p.i. (Figure 1). By 8 h p.i., net replication was observed in all cell lines and could be divided into two distinct phenotypes; epithelial cells with 1–40 bacteria and those containing ≥100 bacteria (Figure 1). Bacteria within this second category we have termed “hyper-replicating” due to their rapid doubling time [18]. The frequency of this phenomenon varied between cell lines, with HeLa and C2Bbe1 cells showing the highest rates. Hyper-replicating *Salmonella* (≥100 bacteria/cell) were evident in 9.2±3.2% of infected HeLa cells, 4.2±2.1% of HuTu 80 cells, 5.1±0.96% of HCT 116 cells and 19±5.9% of C2Bbe1 cells (8 h p.i., Figure 1).

Vacuolar and cytosolic *Salmonella* express a different subset of virulence genes in polarized epithelial cells [18]. Bacteria contained within an SCV are T3SS2-induced, whereas cytosolic *Salmonella* express the two virulence factors necessary for the invasion of non-phagocytic cells, T3SS1 and flagella, hence are induced for an invasive phenotype. To investigate whether this held true for non-polarized epithelial cells, we infected HeLa, HuTu 80, HCT 116 and Caco-2 C2Bbe1 cells with wild type *Salmonella* harboring a destabilized green fluorescent protein (GFP) reporter for T3SS1 gene expression, pMPMA3-*PprgH*-GFP[LVA] [24], fixed at 8 h p.i. and immunostained for flagellin, FliC. Cells whose entire cytosolic space was filled with flagellated bacteria, many of which were GFP-positive, were clearly evident by fluorescence microscopy at 8 h p.i. in all four cell lines (Figure 1). We conclude that hyper-replicating, invasion-primed *Salmonella* are a widespread phenomenon in the cytosol of cultured epithelial cells.

### Vacuolar Escape is Independent of Bacterial Load

**Figure 2:**
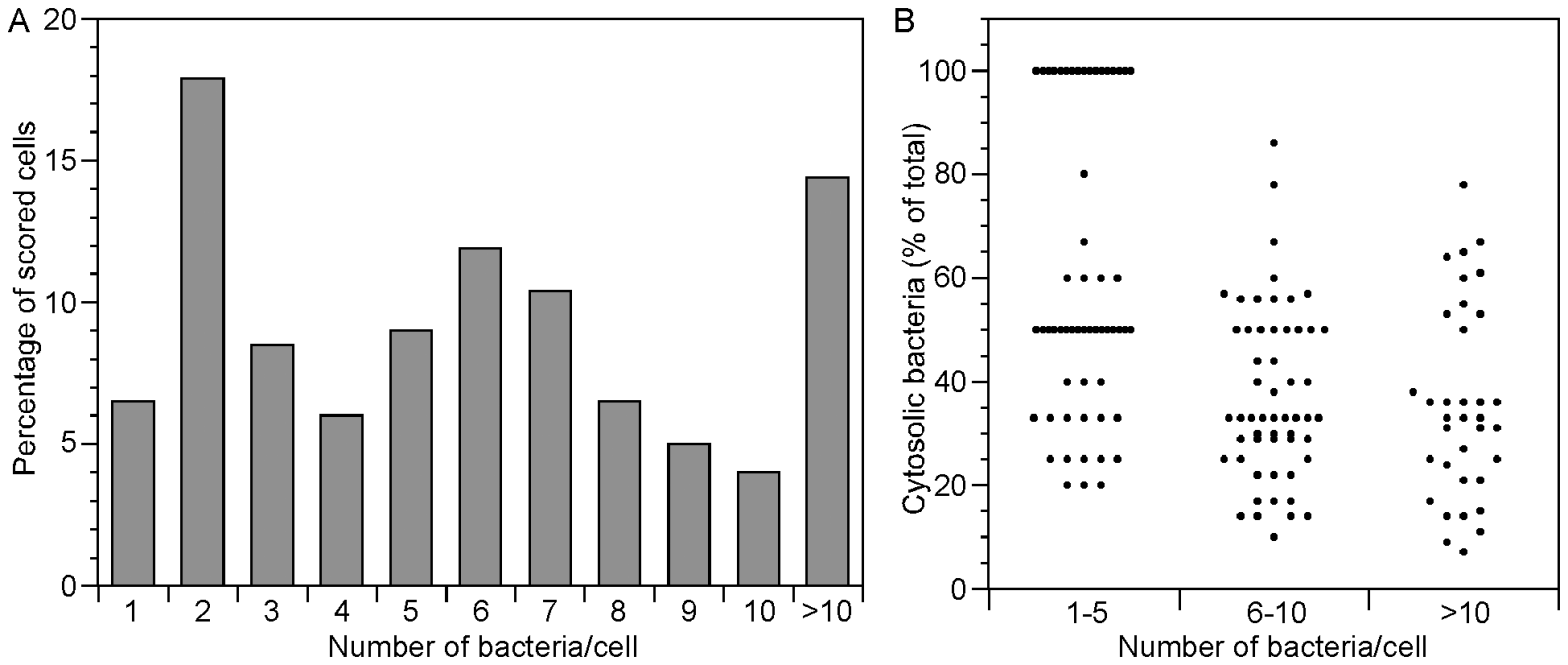
Vacuolar lysis is not dependent upon bacterial load. HeLa cells were infected with mCherry *S*. Typhimurium. At 1 h p.i., the plasma membrane was permeabilized with digitonin, followed by delivery of anti-*Salmonella* LPS antibodies to the cytosol. Monolayers were visualized by fluorescence microscopy. (A) In epithelial cells containing at least one cytosolic bacterium (LPS-positive after digitonin permeabilization), the total number of internalized bacteria was scored. Combined data from three independent experiments (n = 201 cells). (B) In cells containing at least one cytosolic bacterium, the proportion of cytosolic bacteria in the total bacterial load was calculated. Data was binned into three categories: cells containing 1–5 total bacteria, 6–10 total bacteria and >10 total bacteria. Each dot represents one cell. Combined data from two independent experiments (n = 147 cells).

What determines whether or not hyper-replication occurs in an infected epithelial cell? One possibility that we considered is that this could be linked to the number of bacteria initially entering a cell. We observed considerable heterogeneity in the number of internalized bacteria at 1 h p.i. in all epithelial cell lines, from 1 to >10 bacteria/cell (Figure 1), and hypothesized that escape from the nascent vacuole occurs in those cells with higher numbers of internalized bacteria. To test this, HeLa cells were infected with mCherry *Salmonella* and at 1 h p.i. we used the non-ionic detergent, digitonin, to selectively permeabilize the plasma membrane and deliver anti-*Salmonella* lipopolysaccharide (LPS) antibodies directly to the cytosol. The number of bacteria labeled by LPS antibodies was scored by fluorescence microscopy. At 1 h p.i., ∼20% of the internalized bacteria were detected by LPS antibodies, indicating they were free in the cytosol or had a compromised vacuolar membrane. This is in good agreement with previous reports where the proportion of cytosolic *Salmonella* at 1 h p.i. was estimated by the recruitment of autophagy proteins such as LC3, NDP52 and p62 [13], [34]. In cells where at least one bacterium was cytosolic (LPS-positive), we scored the total number of bacteria that had entered that cell. Cytosolic bacteria were detected in cells containing from 1 to >10 bacteria, with the highest frequency occurring in cells containing only two bacteria (17.9%)(Figure 2A). To assess in more detail whether cytosolic release depends upon the initial bacterial load, we calculated the proportion of cytosolic bacteria in cells containing either 1–5 total bacteria, 6–10 total bacteria or >10 total bacteria. The percentage of cytosolic bacteria ranged from 20–100% (1–5 bacteria), 10–86% (6–10 bacteria) and 7–78% (>10 bacteria) (Figure 2B). These data indicate that early vacuolar escape is not affected by the number of bacteria that are internalized into a particular cell, nor is the frequency of vacuolar escape within a cell.

### Vacuolar, but not Cytosolic, Salmonella are Susceptible to Chloroquine

**Figure 3:**
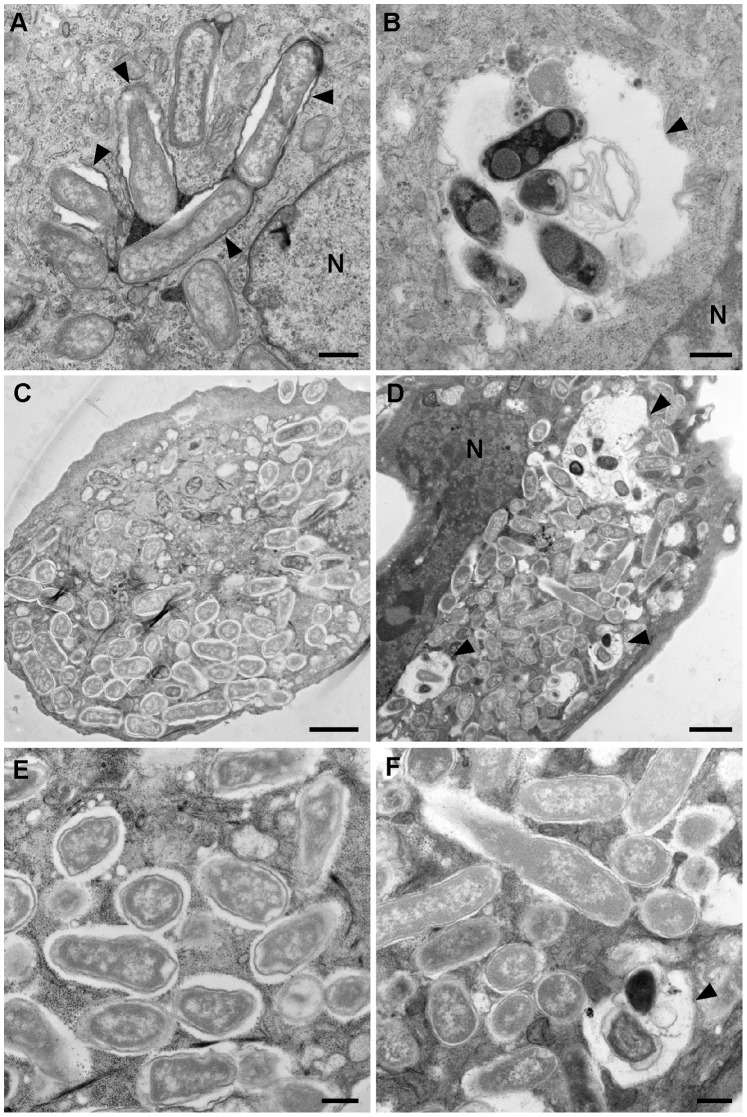
Vacuolar, but not cytosolic, *Salmonella* are susceptible to chloroquine treatment. HeLa epithelial cells were seeded on Thermanox® plastic coverslips and infected with wild type *S*. Typhimurium. At 7 h p.i., cells were left untreated or treated with 400 µM CHQ for 1 h. Untreated and CHQ-treated cells were then fixed at 8 h p.i. and processed for transmission electron microscopy. (A) Vacuolar bacteria in untreated cells. (B) Vacuolar bacteria in CHQ-treated cells. (C) Hyper-replicating, cytosolic bacteria in untreated cells. (D) Hyper-replicating, cytosolic bacteria in CHQ-treated cells. (E) Inset of (C). (F) Inset of (D). Arrowheads indicate bacteria enclosed within vacuoles. N, nucleus. Scale bars are 0.5 µm for (A), (B), (E) and (F), 2 µm for (C) and (D).

*Salmonella* that lyse their nascent vacuole can eventually hyper-replicate in the cytosol of epithelial cells. Between 5–20% of infected epithelial cells harbor hyper-replicating, cytosolic *Salmonella* by 8 h p.i. (Figure 1) [18], [19] but, due to the sheer number of bacteria within these cells, it is not possible to enumerate them by fluorescence microscopy. To precisely determine the proportion of vacuolar and cytosolic bacteria in the entire population, we used an assay that relies upon the differential intracellular distribution of antibiotics in mammalian cells [35], [36]. The weak base chloroquine (CHQ) accumulates to high concentrations within endosomes, but does not access the cytosol [36], and has previously been used to kill *Shigella flexneri* that have failed to lyse their phagocytic vacuole [30], [31]. To validate whether CHQ would preferentially target *Salmonella* within the SCV, we infected HeLa epithelial cells with *S*. Typhimurium SL1344 wild type bacteria and at 7 h p.i., incubated cells with 400 µM CHQ for 1 h. Samples were then processed for transmission electron microscopy (TEM). In the untreated HeLa cells, we observed two distinct phenotypes for intracellular replication: (i) cells were either completely filled with bacteria not enclosed by a vacuolar membrane, but rather were surrounded by an electron-lucent space (Figure 3C, 3E) or (ii) contained lower numbers of bacteria that were in tightly apposed membrane-bound vacuoles (Figure 3A). These two populations had a different fate upon CHQ addition. In cells with few bacteria, large, spacious vacuoles were observed that contained numerous electron-dense bacteria that appeared damaged and/or degraded (Figure 3B). By contrast, the morphology of cytosolic, hyper-replicating *Salmonella* was unaffected by CHQ treatment (Figure 3D, 3F). In some of these cells, we did observe a minor population of bacteria in large, spacious vacuoles, suggesting that some were membrane-bound (Figure 2D, 2F). This agrees with our observations that individual cells can contain both vacuolar and cytosolic bacteria (Figure 2B). Overall, this TEM data implies that CHQ preferentially targets vacuolar *Salmonella*.

**Figure 4:**
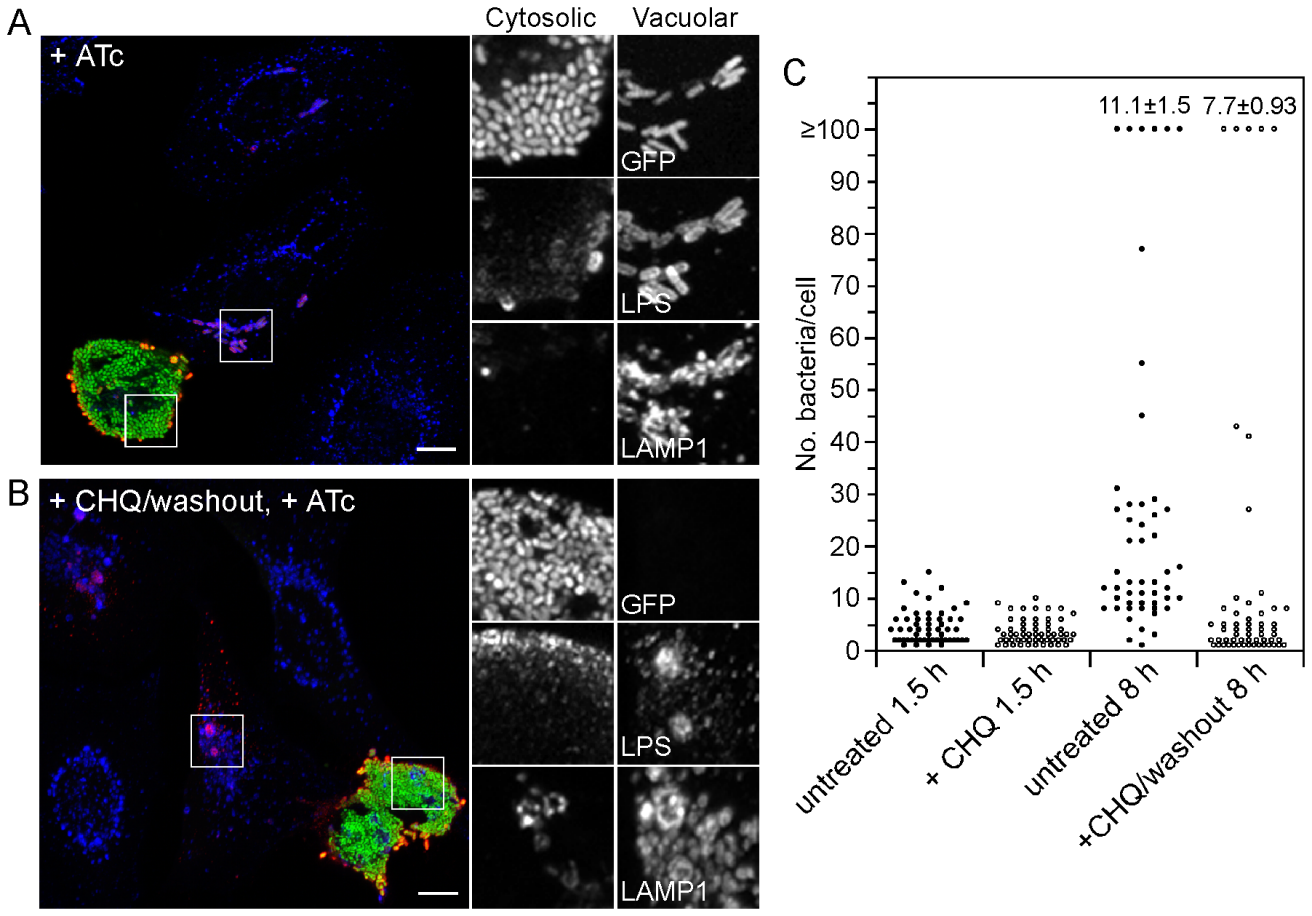
Vacuolar bacteria are transcriptionally inactive and non-replicative after CHQ treatment. (A, B) HeLa epithelial cells were seeded on glass coverslips and infected with wild type *S*. Typhimurium harboring plasmid-borne GFPmut3 under the control of the *tetRA* promoter. At 5 h p.i., cells were treated with 400 µM CHQ for 1 h. CHQ was then washed out and cells were further incubated with 300 ng/ml ATc for 3 h to allow *gfp* transcription. Cells were fixed, permeabilized and immunostained for *Salmonella* LPS (shown in red) and LAMP1 (blue). (A) Confocal image of cells treated with ATc only. Both vacuolar and cytosolic bacteria are GFP-positive. Scale bar is 10 µm. (B) Confocal image of cells treated with CHQ, washed out, then incubated with ATc. Only hyper-replicating, LAMP1-negative bacteria are fluorescent. Scale bars are 10 µm. (C) HeLa cells were infected with mCherry *S*. Typhimurium. Cells were left untreated or incubated with 400 µM CHQ from 30–90 min p.i., then washed out and incubated in CHQ-free growth medium until 8 h p.i. Cells were fixed at 90 min and 8 h p.i. and the number of bacteria per cell scored by fluorescence microscopy. Each dot represents one infected cell. Results are from a representative experiment. The proportion of infected cells containing ≥100 bacteria from three independent experiments (mean ± SD) is shown for each condition at the top of the graph.

To corroborate this, we used an inducible GFP reporter to monitor the viability of vacuolar and cytosolic bacteria after CHQ treatment. HeLa cells were infected with *S*. Typhimurium expressing GFPmut3 under the control of an ATc-inducible promoter, *tetRA*. At 5 h p.i., cells were either left untreated or treated with 400 µM CHQ for 1 h. CHQ was then washed out and cells were incubated for a further 3 h with 300 ng/ml ATc to allow for *gfp* transcription. Cells were fixed, immunostained for LPS and LAMP1 and examined by fluorescence microscopy. In untreated cells, both LAMP1-positive and –negative bacteria exhibited green fluorescence (Figure 4A). Of interest, we noted that anti-LPS antibodies poorly detected hyper-replicating *Salmonella* (Figure 4A inset, 4B inset). Upon CHQ addition and then washout, LAMP1-negative, hyper-replicating bacteria were GFP-positive but vacuolar bacteria were not (Figure 4B). From this data we conclude that only cytosolic bacteria remain transcriptionally active after CHQ treatment.

We further verified the specificity of CHQ for vacuolar bacteria by treating infected cells early after bacterial invasion with CHQ, then monitoring bacterial replication after drug washout. HeLa cells were infected with mCherry *S*. Typhimurium and treated with 400 µM CHQ from 0.5–1.5 h p.i. Drug was then washed out and the incubation continued until 8 h p.i. The number of bacteria per cell was scored by fluorescence microscopy (Figure 4C). At 1.5 h p.i., there was no overt difference in the number of intracellular bacteria in the absence or presence of CHQ. In contrast, CHQ treatment dramatically affected the profile of bacterial replication at 8 h p.i. (Figure 4C). Untreated HeLa cells showed low (1–10 bacteria/cell), moderate (10–80 bacteria/cell) and high (≥100 bacteria/cell) replication phenotypes [19]. Vacuolar replication accounts for low and moderate phenotypes, whereas the high replication phenotype is due to hyper-replicating, cytosolic bacteria. In CHQ-treated cells, only two populations, low and high, were evident at 8 h p.i. The majority of cells contained ≤10 bacteria/cell, similar to what was observed at 1.5 h p.i., suggesting no or minimal vacuolar replication after CHQ treatment. Hyper-replication was still evident after CHQ washout, but at a lower frequency than for untreated cells (Figure 4C), implying that escape from the nascent SCV (≤90 min p.i.) accounts for some, but not all, of the hyper-replication phenotype. From this data we conclude that vacuolar bacteria are compromised by early CHQ treatment, and as a consequence are replication-incompetent, whereas bacteria that escape from the nascent vacuole are not. Collectively, these experiments validate the selectivity of CHQ for vacuolar *Salmonella*.

**Figure 5:**
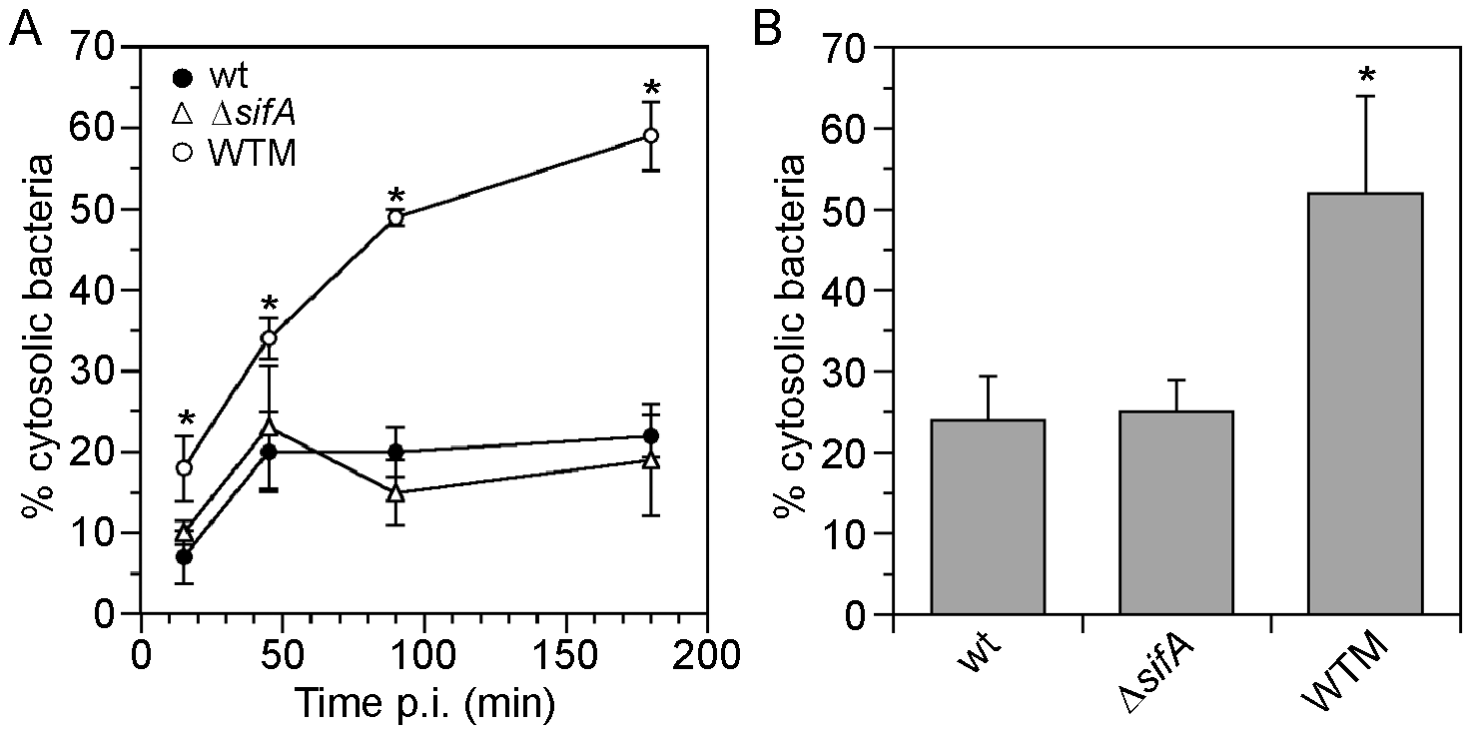
Wortmannin increases the proportion of cytosolic *Salmonella* early after bacterial internalization. (A) Digitonin permeabilization assay. HeLa cells were infected with wild type or Δ*sifA* bacteria. Additionally, cells were pretreated with 100 nM WTM for 45 min prior to infection with wild type bacteria, and inhibitor treatment continued until 90 min p.i., then washed out. The proportion of intracellular bacteria accessible to anti-LPS antibodies delivered to the cytosol was determined at each time point. (B) Cells were infected as in (A) and the proportion of cytosolic bacteria was quantified at 1.5 h p.i. by the chloroquine resistance assay. Data (mean ± SD) are from three independent experiments. Asterisks indicate data significantly different from wild type bacteria, analysis of variance (ANOVA) with Dunnett’s post-hoc analysis, p<0.05.

We next tested whether digitonin permeabilization and/or the CHQ resistance assay could accurately measure changes in the cytosolic accessibility of bacteria. We used two conditions known to increase *Salmonella* replication in the cytosol of epithelial cells: (i) treatment with the phosphoinositide 3-kinase inhibitor, wortmannin (WTM) and (ii) infection with a Δ*sifA* mutant. WTM leads to increased bacterial replication in the cytosol by an unknown mechanism [37], [38]. SifA is a type III effector that is translocated by T3SS2 and required to maintain vacuolar membrane integrity [39]. Δ*sifA* mutants fail to maintain an intact SCV, but only at at late times p.i., and consequently replicate in the cytosol of epithelial cells [17], [39]. First we used digitonin permeabilization of the plasma membrane to deliver anti-*Salmonella* LPS antibodies directly into the cytosol. HeLa cells were infected with mCherry wild type or mCherry Δ*sifA* bacteria, treated with digitonin, followed by incubation with anti-LPS antibodies. The number of bacteria labeled with anti-LPS antibodies was monitored by fluorescence microscopy over a time course of infection (Figure 5A). As early as 15 min p.i., 6.8±2.9% of internalized wild type bacteria were stained with anti-LPS antibodies, indicating they are cytosolic. The proportion of anti-LPS-accessible bacteria increased to 20±4.6% by 45 min p.i. and remained at this level up to 3 h p.i. Treatment with WTM dramatically increased the number of wild type bacteria accessible to LPS antibodies at all time points, such that by 3 h p.i. 59±4.2% of bacteria were cytosolic. Δ*sifA* mutant bacteria were indistinguishable from wild type bacteria over this time period, in agreement with the kinetics of *sifA* expression [39]. Unfortunately, we were unable to utilize digitonin permeabilization together with monoclonal or polyclonal anti-LPS antibodies after 4 h p.i. due to the poor immunodetection of cytosolic, hyper-replicating *Salmonella* (Figure 4A, 4B insets). Total and cytosolic bacteria were also quantified at 1.5 h by gentamicin-protection in combination with the CHQ resistance assay (Figure 5B). Corroborating the digitonin permeabilization results, there was no difference in the proportion of cytosolic wild type and Δ*sifA* bacteria at 90 min p.i. (24±5.4% and 25±3.9%, respectively). However, WTM dramatically increased the percentage of wild type bacteria in the cytosol at 90 min p.i., to 52±12%. These data indicate that WTM affects the frequency of early vacuolar escape and provide proof-of-principle that digitonin permeabilization and CHQ resistance assay can accurately quantify increased numbers of cytosolic *Salmonella*.

### Access to the Cytosol Induces T3SS1 Expression

**Figure 6:**
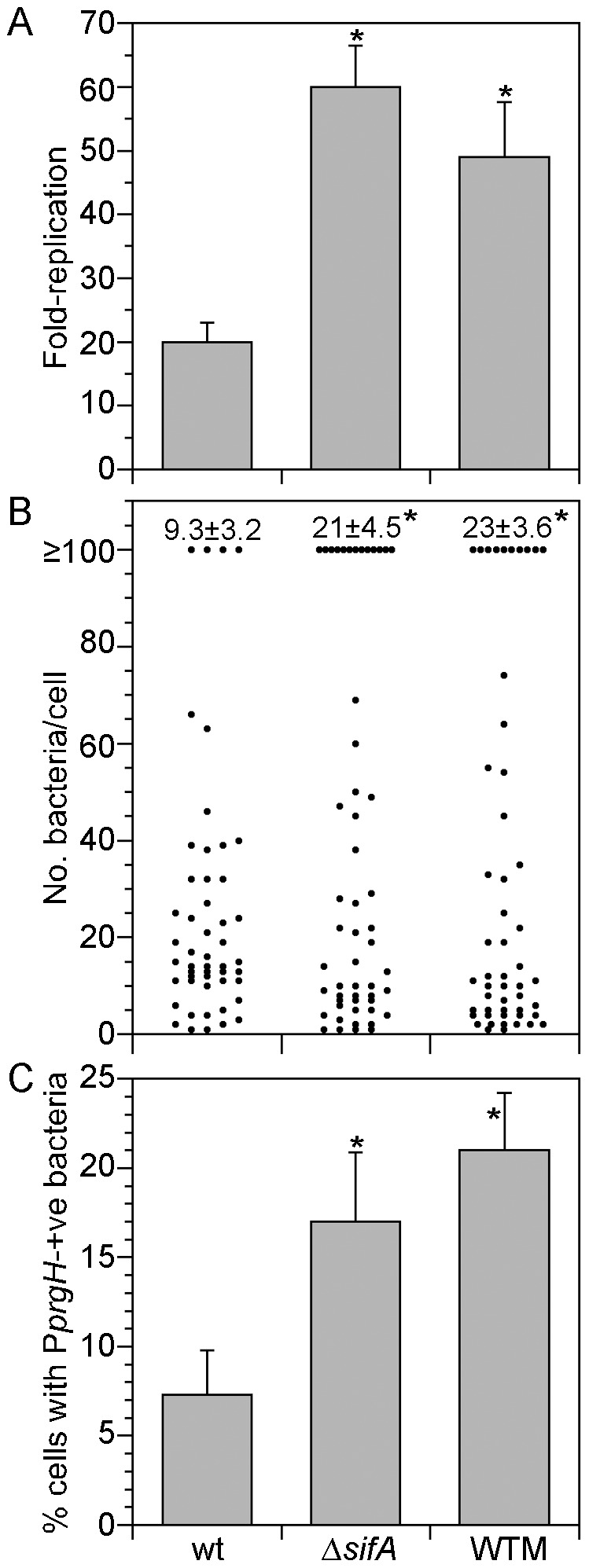
Access to the cytosol induces SPI-1 gene expression. (A) Gentamicin protection assay in HeLa cells. HeLa cells were infected with wild type or Δ*sifA* bacteria. Additionally, cells were pretreated with 100 nM WTM for 45 min prior to infection with wild type bacteria, and inhibitor treatment continued until 90 min p.i., then washed out. Infected cells were solubilized at 1 h and 10 h p.i. and viable bacteria enumerated by plating on LB agar. Fold-replication represents CFU at 10 h p.i. divided by CFU at 1 h p.i. Values (mean ± SD) are from three independent experiments. (B) Cells were infected as described above with mCherry wild type or mCherry Δ*sifA* mutant bacteria. At 10 h p.i., cells were fixed and the number of bacteria per cell scored by fluorescence microscopy. Each dot represents one infected cell. Data are from a representative experiment. The proportion of infected cells containing ≥100 bacteria from three independent experiments (mean ± SD) is shown for each condition at the top of the graph. (C) Cells were infected as described above with wild type or Δ*sifA* bacteria harboring a GFP reporter plasmid for SPI-1 activity, pMPMA3ΔPlac-P*prgH*-GFP[LVA]. At 10 h p.i., cells were fixed and bacteria immunostained with anti-LPS antibodies. The frequency of infected cells containing at least one GFP-positive bacterium was scored by fluorescence microscopy. Data are from three independent experiments (mean ± SD). wt, wild type bacteria; Δ*sifA*, Δ*sifA* mutant bacteria; WTM, wild type bacteria plus WTM treatment. Asterisks indicate data significantly different from wild type bacteria, analysis of variance (ANOVA) with Dunnett’s post-hoc analysis, p<0.05.

Hyper-replicating, cytosolic *Salmonella* are flagellated and express T3SS1 at later times in epithelial cells [18]. Here we have shown that WTM treatment increases the proportion of cytosolic bacteria early after bacterial internalization (Figure 5). By contrast, Δ*sifA* bacteria undergo increased vacuole lysis only at late stages in SCV biogenesis (>6 h p.i.; [39]). We used these two infection conditions to determine whether the timing of vacuolar escape influences T3SS1 induction in cytosolic *Salmonella*. HeLa epithelial cells were infected with wild type bacteria in the absence or presence of WTM from 45 min pre-infection until 90 min p.i. or with Δ*sifA* mutant bacteria. Cells were then solubilized at 1 h and 10 h p.i. for enumeration of viable bacteria. Similar to previous reports [17], [37], [38], infection with Δ*sifA* mutant bacteria and WTM treatment both led to increased levels of replication (Figure 6A; wild type bacteria 20±3.0-fold, wild type bacteria plus WTM 49±8.7-fold and Δ*sifA* mutant bacteria 60±6.5-fold). We next used single-cell analysis to investigate this net increase in replication. Cells were infected with mCherry *S*. Typhimurium and scored by fluorescence microscopy for the number of bacteria per cell at 10 h p.i. (Figure 6B). For wild type infection, 9.3±3.2% of cells contained ≥100 bacteria/cell. This proportion increased for both Δ*sifA* bacteria (21±4.5%) and wild type bacteria plus WTM treatment (23±3.6%). Therefore, the net increase in replication at 10 h for WTM treatment and Δ*sifA* bacteria can be attributed to an increase in the percentage of infected cells that harbor hyper-replicating bacteria.

We then assessed the relationship between the timing of vacuolar rupture and SPI1 expression in cytosolic bacteria. HeLa cells were infected with wild type or Δ*sifA* bacteria harboring a fluorescent reporter for the SPI1 regulon, pMPMA3ΔPlac-P*prgH*-GFP[LVA] [24]. Cells infected with wild type bacteria were left untreated or treated with WTM until 90 min p.i., then washed out. At 10 h p.i. cells were fixed and immunostained with anti-LPS antibodies to detect *Salmonella*. The number of infected cells that contained at least one GFP-positive bacterium was scored by fluorescence microscopy (Figure 6C). There was a 2.9-fold increase in the fraction of infected cells with SPI-1 induced wild type bacteria in the presence of WTM, from 7.3±2.5% to 21±3.2%. Likewise, there was a 2.3-fold increase in the proportion of SPI1-induced bacteria for the Δ*sifA* mutant (17±3.9%). Collectively, this data shows that regardless of whether *Salmonella* escapes early or late from the SCV, access to the cytosol is sufficient to induce expression of the SPI1-encoded gene, *prgH*.

### Kinetics of Salmonella Access to Epithelial Cell Cytosol

**Figure 7:**
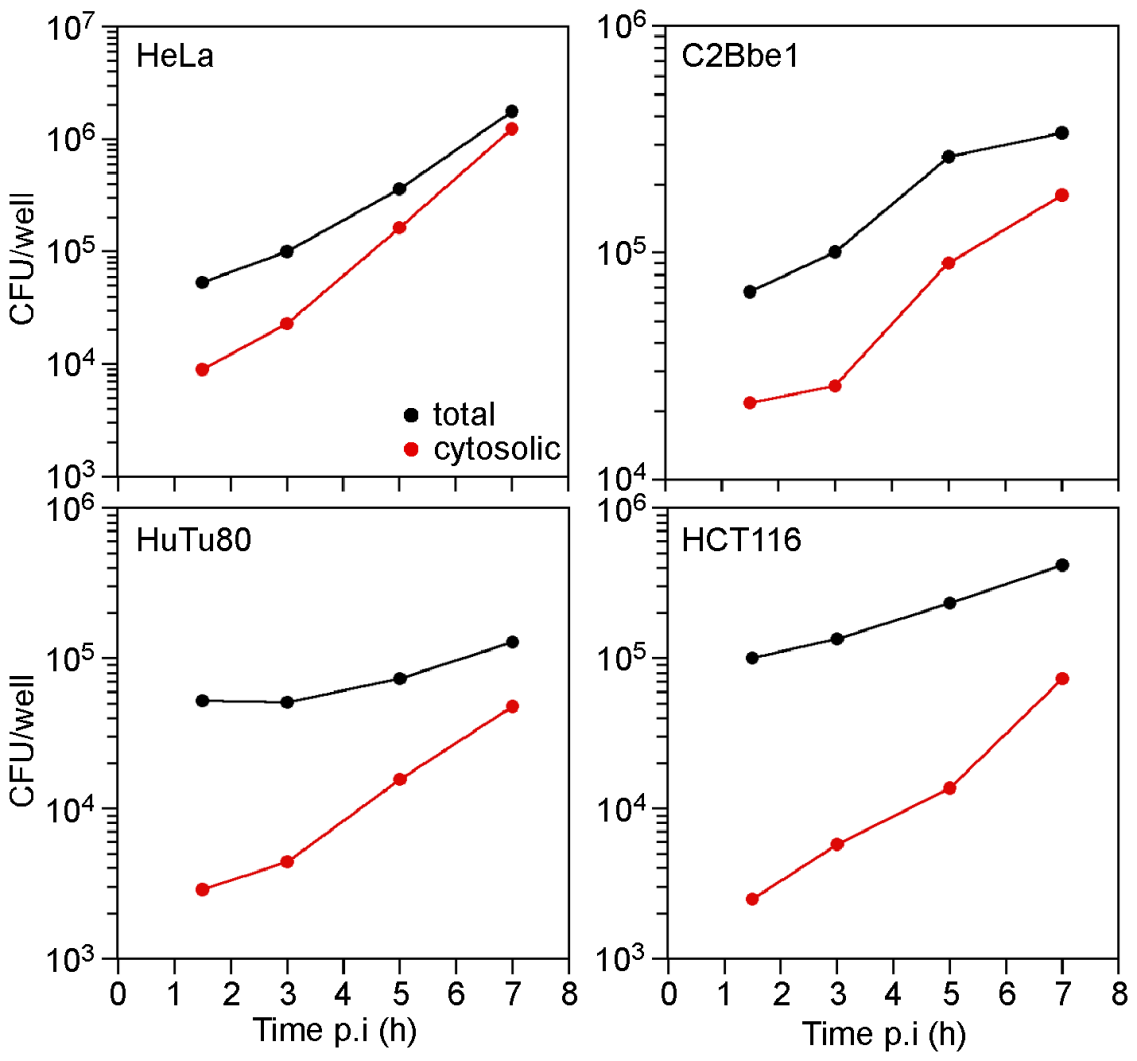
Time course of cytosolic replication in epithelial cell lines. Epithelial cells (HeLa, Caco-2 C2Bbe1, HuTu 80 and HCT 116) were seeded in 24-well plates and infected with wild type *S*. Typhimurium. One hour prior to each time point, two wells were treated with CHQ. At the indicated time, duplicate untreated (total CFU, black dots) and duplicate CHQ-treated cells (cytosolic CFU, red dots) were solubilized and serial dilutions plated on LB agar for CFU enumeration. Results are representative of at least three independent experiments.

We have shown that hyper-replicating *Salmonella* are present in numerous epithelial cell lines, albeit with different frequencies (4.2 - 19% of infected cells at 8 h p.i., Figure 1), which could impact their contribution to the total population. To assess this, we quantified the proportion of cytosolic bacteria in the total bacterial population over a time course of infection in these cell lines. Towards this aim, epithelial cells were infected with wild type *S*. Typhimurium and total and cytosolic bacteria were quantified by gentamicin protection and CHQ-resistance assays, respectively, at 1.5, 3, 5 and 7 h p.i. (Figure 7). For all cell lines the number of total bacteria increased with time, in agreement with single-cell analyses (Figure 1). Likewise, more bacteria were present in the cytosol at 7 h p.i. compared to 1.5 h p.i., suggesting net replication in the cytosol or continual escape from the SCV with time. Initial vacuolar lysis was most frequent in HeLa and Caco-2 C2Bbe1 cells, with more than 20% of bacteria in the cytosol by 1.5 h p.i. (Figure 7, Table 1). By comparison, less than 10% of the internalized bacteria were present in the cytosol of HCT 116 and HuTu 80 cells at 1.5 h p.i. (Figure 7, Table 1). Independent assessment of the percentage of cytosolic bacteria using digitonin permeabilization concurred with data from the CHQ resistance assay at 1.5 h p.i. (Table 1). By 7 h p.i., approximately half of the bacterial population was free in the cytosol of HeLa and Caco-2 C2Bbe1 cells (Figure 7, Table 1). Lower frequencies of cytosolic bacteria were quantified in HCT116 and HuTu80 cells at 7 h p.i., in accordance with comparatively fewer bacteria lysing the nascent SCV (Figure 7, Table 1). Overall, these observed frequencies are in agreement with the relative proportion of cells containing ≥100 bacteria/cell for each cell line (Figure 1). Altogether, these results identify cytosolic *Salmonella* as a significant proportion of the total bacterial population, especially at later times of infection.

**Table 1:** Quantification of cytosolic *Salmonella* in different epithelial cell lines.

Epithelial cell line	Cytosolic bacteria at 90 min p.i. (% total)		Cytosolic bacteria at 7 h p.i. (% total)
	CHQ resistance assay	Digitonin permeabilization	CHQ resistance assay
HeLa	22±8.9	20±4.9	51±5.9
Caco-2 C2Bbe1	27±6.7	N.D.	45±9.8
HCT 116	5.8±3.2	6.8±3.7	15±3.8
HuTu 80	7.5±4.9	12±2.8	33±3.2

Cells were infected with wild type bacteria (CHQ resistance assay) or mCherry wild type bacteria (digitonin permeabilization). The percentage of cytosolic bacteria in the total population was quantified by these two assays at the indicated times. Data are mean ± SD from at least three independent experiments. N.D., not determined.

### SPI1 Contributes to Vacuole Lysis and Replication in the Cytosol

Efficient entry of *Salmonella* into non-phagocytic cells such as epithelial cells requires T3SS1 and its cognate type III effectors [40]–[42]. Over the duration of our experiments we noted that there was a wide range in the permissiveness of different epithelial cell lines to entry by a ΔSPI1 mutant, as assessed by gentamicin protection assay (Table 2). This mutant is lacking the entire *Salmonella* pathogenicity island-1 (SPI-1) genetic region, encoding for the T3SS1 apparatus, regulatory proteins and T3SS1 effectors. For example, we found that HT-29 cells, derived from a human colorectal adenocarcinoma, were the least permissive of the tested cell lines (Table 2). At the other end of the spectrum, another colorectal adenocarcinoma cell line, Caco-2 C2Bbe1, was 500-fold more permissive to entry of a ΔSPI1 mutant (Table 2). This is in agreement with the reported T3SS1-dependent and –independent invasion processes for *S*. Typhimurium entry of fibroblasts and kidney epithelial cells [43], [44]. Of note, *S.* Typhimurium has been shown to bind specific carbohydrate epitopes present on the surface of a subpopulation of Caco-2 C2Bbe1 cells [45], which might contribute to the observed T3SS1-independent entry mechanism.

**Table 2:** Entry of a ΔSPI1 mutant into different epithelial cell lines.

Epithelial cell line	Characteristics	Invasion rate (% of wild type bacteria)
HT-29	Colorectal adenocarcinoma	0.0046±0.0045
HuTu 80	Duodenal adenocarcinoma	0.041±0.020
HCT 116	Colorectal carcinoma	0.056±0.010
SW480	Colorectal adenocarcinoma	0.065±0.022
HeLa	Cervical adenocarcinoma	0.23±0.08
Caco-2 C2Bbe1	Colorectal adenocarcinoma	2.4±0.69

Bacterial entry was quantified by gentamicin protection assay at 1 h p.i. The invasion rate of the ΔSPI1 mutant was compared to wild type bacteria for each cell line. Data are means ± SD from at least three independent experiments.

**Figure 8:**
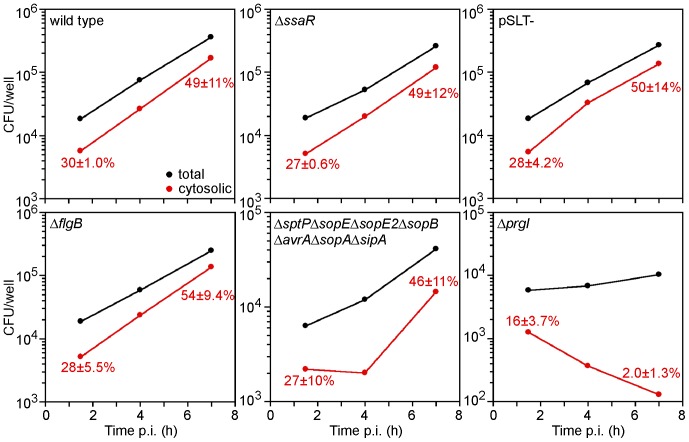
SPI-1 is required for vacuole lysis and proliferation in the cytosol. Caco-2 C2Bbe1 cells were infected with the following *S*. Typhimurium strains; SL1344 wild type, Δ*ssaR* (defective for T3SS2 assembly), pSLT- (virulence-plasmid cured), Δ*flgB* (defective for flagellar assembly), Δ*sptP*Δ*sopE*Δ*sopE2*Δ*sopB*Δ*avrA*Δ*sopA*Δ*sipA* (“effectorless” mutant), Δ*prgI* (defective for T3SS1 assembly). CHQ was added for 1 h prior to each time point. At the indicated times, untreated and CHQ-treated monolayers were solubilized and plated on LB agar for CFU enumeration. Total bacteria are shown by black dots, CHQ-resistant bacteria (cytosolic) by red dots. Results are representative of at least three independent experiments.

We took advantage of the susceptibility of Caco-2 C2Bbe1 cells to T3SS1-independent entry to investigate the contribution of known bacterial virulence factors, including SPI-1, to vacuolar lysis and cytosolic replication in epithelial cells. Bacterial strains tested were Δ*ssaR* (defective for T3SS2 assembly and translocation), χ3340 (pSLT^−^, cured of the virulence plasmid), Δ*flgB* (defective for flagellar apparatus assembly), Δ*prgI* (defective for T3SS1 assembly and translocation) and an “effectorless” mutant (deleted for seven effectors delivered by T3SS1). Caco-2 C2Bbe1 cells were infected with wild type and mutant bacteria and total and cytosolic bacteria were determined over a time course by gentamicin protection and CHQ resistance assays, respectively. Profiles of Δ*ssaR*, χ3340 (pSLT^−^) and Δ*flgB* bacteria were comparable to wild type bacteria over the timecourse (Figure 8), indicating that neither T3SS2, genes encoded on the virulence plasmid nor the flagellar apparatus contribute to lysis of the vacuole or bacterial replication in the cytosol. Similarly, the proportion of cytosolic bacteria for the “effectorless” mutant was not statistically different from wild type bacteria at 1.5 h and 7 h p.i. (Figure 8), excluding a role for *sptP*, *sopE*, *sopE2*, *sopB*, *avrA*, *sopA* or *sipA* in either of these processes. By contrast, the T3SS1 mutant, Δ*prgI*, showed a dramatically altered profile for both total and cytosolic bacteria (Figure 8). Similar results were observed for two other SPI1 null mutants, Δ*invA* and ΔSPI1 (Figure S1). Overall, there was minimal bacterial replication for this mutant in Caco-2 C2Bbe1, in agreement with our previous findings that SPI-1 is required for intracellular proliferation in HeLa cells [38]. Furthermore, this mutant was affected for SCV lysis and cytosolic survival. The T3SS1 apparatus has been shown to “damage” the nascent SCV [46], leading to bacterial recognition by autophagy [13]. In agreement, we found significantly fewer *prgI* mutant bacteria were in the cytosol at 1.5 h p.i. (Figure 7). However, an additional mechanism must contribute to lysis of the nascent SCV because vacuolar release for the *prgI* mutant was reduced by only half compared to wild type bacteria. This mutant was severely defective for cytosolic proliferation/survival, with only 2.0±1.3% of the total population of *prgI* bacteria present in the cytosol at 7 h p.i. compared to 49±11% for wild type bacteria. Collectively, these results impart a role for T3SS1 and another unidentified mechanism in early escape from the SCV, and T3SS1 in colonization of the cytosol in epithelial cells, excluding the T3SS1-translocated effectors *sptP*, *sopE*, *sopE2*, *sopB*, *avrA*, *sopA* and *sipA*.

## Discussion

While *S*. Typhimurium has traditionally been categorized as a vacuolar pathogen, our data advocate that it also be considered as a cytosolic bacterium in epithelial cells. In fact, the distinction between whether a pathogen is “vacuolar” or “cytosolic” is becoming blurred. For example, the historical view is that *M. tuberculosis*, the causative agent of human tuberculosis, resides within a membrane-bound vacuole that does not fuse with lysosomes. Recently, this was challenged by several independent studies [47]–[50]. In human and mouse macrophages it was shown that pathogenic mycobacteria, but not non-pathogenic species, can translocate from a vacuole to the cytosol as early as 3 h p.i. [47]–[49]. The frequency of vacuolar rupture was estimated to be 25–80% of total bacteria, depending upon the infecting mycobacterial species [48] and bacterial access to the cytosol required ESAT-6, which is secreted by a type VII secretion system, ESX-1 [47]–[49]. In another example, the facultative intracellular pathogen, *Francisella tularensis*, is regarded as a highly adapted cytosolic bacterium [51]. Shortly after its entry into host cells, *F. tularensis* lyses its phagosome and then replicates extensively in the cytosol. However, in mouse macrophages some bacteria re-enter the endocytic compartment around 24 h p.i. via an autophagy-dependent process [33], although the role for this vacuolar shift remains unknown. Together with our work here on *S*. Typhimurium, these studies illustrate that it is not always feasible to categorize the intracellular lifestyle of a pathogen as being either vacuolar or cytosolic.

Application of digitonin permeabilization and the CHQ resistance assay has allowed for the quantification of cytosolic *Salmonella* for the first time. By 90 min p.i., ∼20% of bacteria are cytosolic in epithelial cells. Compared to other intracellular pathogens that lyse their phagocytic vacuole, this proportion is comparatively low. For example, the CHQ resistance assay estimates that between 60–70% of *S. flexneri* are cytosolic at 90 min in HeLa [52] and 80% by 75 min p.i. in J774 cells [31]. Using digitonin permeabilization to deliver anti-LPS antibodies, it has been shown that 80% of *F. tularensis* have lysed their vacuole by 60 min in murine bone-marrow derive macrophages [53]. For the Gram-positive bacterium, *Listeria monocytogenes*, a phalloidin binding assay has typically been used to detect cytosolic bacteria via their association with F-actin [54]. This assay indicates that 56–85% of *Listeria* are cytosolic by 90 min in J774 mouse macrophage-like cells [54], [55]. Despite their less frequent vacuolar escape, the ability of *Salmonella* to replicate faster in the epithelial cytosol compared to the SCV leads to there being up to half of the population free within the cytosol at later times. We, and others, have also noted the presence of cytosolic *Salmonella* in epithelial cells *in vivo* [18], [56], although we have not yet been able to accurately quantify their prevalence.

We have shown here that CHQ preferentially targets vacuolar *Salmonella*. A similar affinity for vacuolar, but not cytosolic, *S. flexneri* has also been reported [30], [31]. We are currently unsure as to how CHQ acts upon these intravacuolar pathogens. For the Gram-positive bacterium, *Bacillus megaterium*, CHQ inhibits DNA and RNA biosynthesis [57]. Despite reports of CHQ inhibiting the exponential phase growth of *Escherichia coli* in broth [58], CHQ had no antibacterial activity on exponentially growing *S*. Typhimurium in complex broth (LB-Miller) or minimal media (low phosphate, low magnesium pH 5.8 [59]) at concentrations up to 10 mM (our unpublished results). CHQ is a weak base that increases intralysosomal pH [60], [61]. Phagolysosomal alkalinization has been shown previously to enhance antibiotic efficacy [61]–[64] and limit iron availability [65], leading to increased bacterial killing in the phagosome. One possibility is that a CHQ–induced elevation of the SCV lumenal pH improves the antimicrobial activity of gentamicin, which normally is quite limited at an acidic pH [66]. However, gentamicin protection assays in the presence of another weak base, ammonium chloride, did not lead to the intravacuolar killing of *Salmonella* (our unpublished results), suggesting that vacuolar alkalinization is not the sole explanation. Despite not knowing the mechanism of action of CHQ, we have validated its predilection for vacuolar bacteria and believe that the CHQ resistance assay will be a useful tool for delineating bacterial and host determinants of the cytosolic lifestyle of *Salmonella*.

Autophagy is an important host defense mechanism against intracellular microbes [67]. Autophagy-associated proteins, such as LC3, p62 and NDP52, are recruited to *Salmonella* that lyse their nascent vacuole, with maximal association at 1 h p.i. in HeLa cells [13], [68], [69]. Ubiquitination of cytosolic bacteria peaks later, at 4 h p.i. [13]. Knockdown of the autophagy-associated receptor proteins p62, NDP52 and optineurin, which recognize ubiquitinated bacteria, leads to an enhanced proliferation of *Salmonella* in HeLa cells [70]. Collectively, from these observations it has been concluded that autophagy: (i) restricts the cytosolic growth of *Salmonella* and (ii) is required for the clearance of cytosolic *Salmonella* [13], [68]–[70]. However, our data show that cytosolic *Salmonella* are not eliminated in epithelial cells. In fact, more than half of the total population are cytosolic by 7 h p.i., suggesting that some of the bacteria that escape from their initial phagosome can evade autophagic recognition in order to hyper-replicate in the cytosol. Additionally, a Δ*sifA* mutant hyper-replicates in the cytosol of epithelial cells upon the loss of its vacuolar membrane integrity (Figure 6) [17] and is not targeted by autophagy ≥6 h p.i. [71]. Recent work from Tattoli et al. [72] also illustrates that autophagic recognition of *Salmonella* is only a transient response, and bacterial re-activation of the mTor signaling pathway prevents autophagic targeting of bacteria after 4 h p.i. To incorporate all of these observations, we propose that autophagic control of *Salmonella* is not absolute, and autophagic recognition is temporally limited i.e. basal levels of autophagy are unable to adequately control the cytosolic proliferation of *Salmonella* after escape from the nascent vacuole and *Salmonella* can prevent their recognition by autophagy once they are hyper-replicating in the cytosol. Our data alludes to SPI-1 being required for survival of *Salmonella* in the epithelial cytosol and future studies are underway to address the possible mechanisms.

## Supporting Information

Figure S1 **Phenotype of SPI-1 null mutants in the CHQ resistance assay.** Caco-2 C2Bbe1 cells were infected with S. Typhimurium *invA*::kan or ΔSPI1::kan mutants. CHQ was added for 1 h prior to each timepoint. At the indicated times, untreated and CHQ-treated monolayers were solubilized and plated on LB agar for CFU enumeration. Total bacteria are shown by black dots, CHQ-resistant bacteria (cytosolic) by red dots. Results are representative of at least three independent experiments.
(TIF)
