# Supplementary figures and images for: Quantitative Assessment of Cytosolic Salmonella in Epithelial Cells
Source: PLoS One. 2014 Jan 6;9(1):e84681. doi: 10.1371/journal.pone.0084681 (PMC3882239; doi:10.1371/journal.pone.0084681)

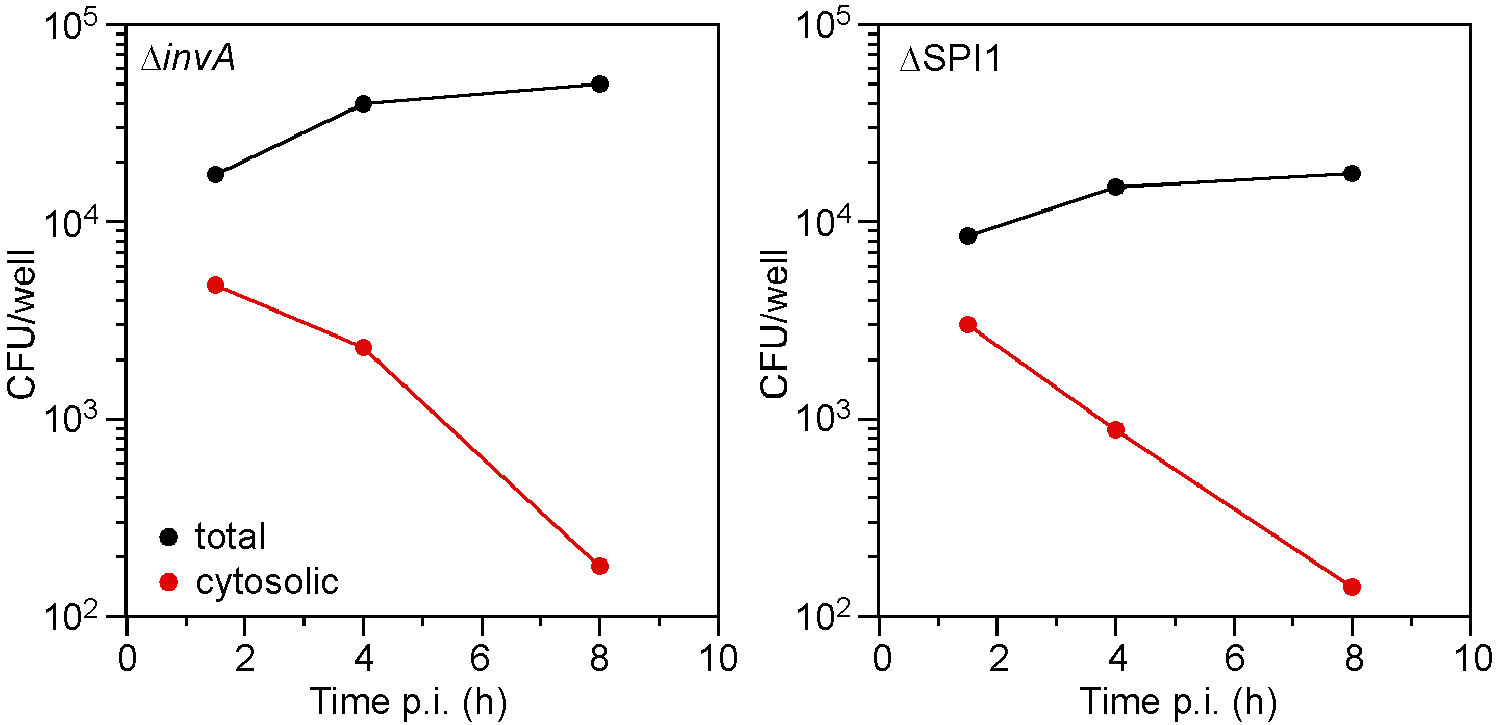

Supplement: Figure S1 — Phenotype of SPI-1 null mutants in the CHQ resistance assay. Caco-2 C2Bbe1 cells were infected with S. Typhimurium invA::kan or ΔSPI1::kan mutants. CHQ was added for 1 h prior to each timepoint. At the indicated times, untreated and CHQ-treated monolayers were solubilized and plated on LB agar for CFU enumeration. Total bacteria are shown by black dots, CHQ-resistant bacteria (cytosolic) by red dots. Results are representative of at least three independent experiments. (TIF) [file pone.0084681.s001.tif]
